# Culinary Medicine or Culinary Nutrition? Defining Terms for Use in Education and Practice

**DOI:** 10.3390/nu16050603

**Published:** 2024-02-22

**Authors:** Sharon Croxford, Emma Stirling, Julia MacLaren, John Wesley McWhorter, Lynn Frederick, Olivia W. Thomas

**Affiliations:** 1 Melbourne Campus, School of Behavioural and Health Sciences, Faculty of Health Sciences, Australian Catholic University, Fitzroy, VIC 3065, Australia; emma.stirling@acu.edu.au; 2 Alberta Health Services, Calgary, AB T3M 1M4, Canada; julia.maclaren@albertahealthservices.ca; 3 Suvida Healthcare, Houston, TX 77027, USA; wmcwhorter@suvidahealthcare.com; 4 FamilyCook Productions, New York, NY 10017, USA; lynn@familycookproductions.com; 5 Boston Medical Center, Boston, MA 02118, USA; olivia.thomas@bmc.org

**Keywords:** culinary nutrition, culinary medicine, teaching kitchen, interprofessional education (IPE), interprofessional practice (IPP)

## Abstract

Examination of how terms such as culinary nutrition, culinary nutrition science, culinary medicine, culinary nutrition professional, culinary nutrition intervention, culinary nutrition activity, and culinary nutrition competency are used in practice, and the creation of consensus definitions will promote the consistent use of these terms across work areas and disciplines. Thirty leading practitioners, academics, and researchers in the fields of food and nutrition across Australia, the United States, Canada, United Kingdom, Europe, and Asia were approached by investigators via email to submit definitions of key terms using a Qualtrics survey link. Further participants were reached through snowball recruitment. Initial emails were sent in October and November 2021 with subsequent reminders between November 2021 and March 2022. Two researchers undertook content analysis of the text answers for each of the terms and generated definitions for discussion and consensus. Thirty-seven participants commenced the survey and twenty-three submitted one or more definitions. Agreed definitions fell into two categories: practice concepts and practitioners. Further discussion amongst investigators led to the creation of a visual map to demonstrate the interrelationship of terms. Culinary nutrition science underpins, and interprofessional collaboration characterizes practice in this area, however, further work is needed to define competencies and model best practice.

## 1. Introduction

There is increasing evidence that cooking and related skills and knowledge is associated with a healthier diet, overall health, and social determinants [1,2]. In addition, there is growing evidence that home cooking is in decline, with calls to invest in culinary interventions (cooking classes or related activity) to improve population health [3].

Culinary interventions with consumers or population groups have a long history of use in different settings and by professionals or lay people from a range of backgrounds, training, and competencies. Examples include school kitchen garden programs delivered by cooks and parent volunteers, secondary school hospitality and home economics curriculum delivered by teachers, primary school interventions delivered by school dietitians, fine dining masterclasses delivered by chefs, cooking programs designed and delivered by home economists, and cooking classes in community-based venues run by volunteers, migrant community members, or chefs. There may or may not be an emphasis on health or healthy cooking during these programs [4]. Evaluation and research have led to evidence-based guidelines and recommendations on best practice culinary interventions [5,6]. In addition, research in this field is demonstrating evidence of a wide range of positive impacts and outcomes from cooking classes, depending on the scope such as increased family cohesiveness [7] and improved household food budgeting [2].

In the last ten years, there has been a growing interest in food and nutrition-related health care provided as health-related culinary interventions, designed, and delivered by qualified health professionals independently or as part of interprofessional teams [8]. High patient engagement and multifactorial psychosocial support is required for complex behavioral modification [9]. Health-related culinary interventions harness experiential, practical, and skills-based intervention strategies to achieve sustained dietary changes [10]; the emerging literature is demonstrating benefits to health outcomes [2,11]. This type of intervention is often described as ‘culinary medicine’ or ‘culinary nutrition’ and is conducted in person or virtually from a teaching kitchen [12]. There are various established delivery models including cookalongs, demonstrations, pre-recorded content, counselling, e-consults [13], and in-home 1:1 cooking coaching. These interventions often complement traditional health care and can be structured as a shared health care model with multidisciplinary and interprofessional teams including doctors, dietitians, occupational therapists, and nurses. Practitioners are partnering with chefs, health coaches, nutritionists, and other paraprofessionals to deliver these interventions.

### 1.1. Medical Professionals

There is new emphasis for competent medical professionals to design and deliver health-related culinary interventions facilitated independently or in collaboration with culinary and/or nutrition experts [14]. Teaching kitchens and cooking classes also have a history of use, to varying degrees, in educating health and medical students on nutrition-related curriculum [15,16]. There are now over 34 medical schools in the United States using a teaching kitchen model with a nutrition curriculum for medical students [15].

### 1.2. Dietitians

Dietitians, referred to as Registered Dietitian Nutritionists (US), Registered Dietitians (Canada), and Accredited Practising Dietitians (Australia), are accredited and registered professional food and nutrition experts. In clinical settings, dietitians provide Medical Nutrition Therapy, which includes nutrition screening, diagnostics, therapy, care planning, and counselling services for the purpose of disease management [17]. In addition, dietitians are trained in relative competencies in health-related food and nutrition activities such as recipe development, nutrient analysis, menu design and review, ingredient procurement, food sustainability, and culturally safe practice [18].

Since the origins of the nutrition and dietetics professions more than eighty years ago, the training of skills in culinary techniques is evident with food service management being a stated domain in historical competencies standards [19]. Furthermore, the U.S. Academy of Nutrition and Dietetics Food and Culinary Professionals Dietetic Practice Group (FCPDPG) identified 11 core competencies in 2015 as a recommendation to dietetic educators to include more food and culinary knowledge into training programs [20]. Given this historical integration of food service management and the recent recommendations, dietitians are uniquely positioned to help lead the advancement of culinary nutrition and culinary medicine by integrating their specialized nutrition knowledge with culinary skills to improve patient outcomes [21]. As a heath profession with codes of conduct, dietitians/nutritionists also ensure quality, safe, evidence-based practice in culturally safe and respectful ways for the health and well-being of people [22].

The inherent competencies embedded in the training of every dietitian position them among health care providers to practice ‘culinary nutrition’ or ‘culinary medicine’ and provide food, nutrition, and culinary interventions [23]. Dietitians facilitate food and nutrition education in public health programs, community health care settings, primary care settings, secondary and tertiary health care settings as well as staff wellness and food retail applications [21]. Given the growing body of evidence, accredited dietetic practice programs and postgraduate qualifications have started to integrate culinary training into their curricula [24,25,26]. Despite the growing adoption of culinary training in food and nutrition education, standardized ‘culinary nutrition’ training has yet to be formally incorporated into competency standards [27].

### 1.3. Other Health and Paraprofessionals

Interest in food, culinary and nutrition education, and the teaching kitchen model is also evident in other health and related disciplines, frequently as interprofessional practice (IPP) or interprofessional education (IPE). Occupational, rehabilitation, recreational, mental health, and speech and language therapists often practice through food or kitchen-related tasks (as cooking is a familiar task of daily living) and now deliver multidisciplinary programs for patients [1]. Nutritionists and nutrition science researchers with tertiary nutrition science qualifications are involved in community and public health cooking education, often with a focus on food literacy and food security [28]. Interprofessional ‘culinary medicine’ education has been established for students in pharmacy, social work, nursing, and dentistry schools [29,30]. Furthermore, a wide variety of health care professionals including public health practitioners, psychologists, physician assistants, and physiotherapists in over 80 countries engaged in a free, online introductory resource on ‘culinary medicine’ [31].

### 1.4. Chefs and the Culinary Arts

In culinary arts education, there is an established teaching of nutrition concepts, following the belief that nutrition knowledge is important for chefs [32], and recommendations to underscore the importance of ‘culinary nutrition’ in gastronomy [33]. Existing content areas include healthy recipe and menu development often aligned with country-specific dietary guidelines, foods for special diets including food allergy management, and religious and culturally specific diets [34]. There are professional development initiatives to upskill qualified chefs in nutrition-related content such as universal food allergen training [35]. Some individual chefs are furthering their careers with dual qualifications in nutrition and dietetics [36], and certified culinary professionals can gain associate membership of the Academy of Nutrition and Dietetics [37]. Specialty postgraduate pathways for chefs in the culinary arts in applied nutrition are also emerging [38].

### 1.5. Defining Terms

As the field of health-related culinary interventions conducted by health professionals or interprofessional teams continues to grow, there is a need to define terms. It is evident that terms such as ‘culinary nutrition’, ‘culinary medicine’, and ‘culinary nutrition competency’ are used in practice, education, and throughout the literature. Examination of how these terms is used by practitioners, academics, and researchers, and the creation of consensus definitions will promote the consistent use of these terms across work areas and disciplines. This will help drive consistency in education, research, and practice, further define and protect the appropriate scope of practice, and importantly, distinguish health-related culinary interventions from cooking classes of a general nature.

### 1.6. Aim

The aim of this study was to investigate how common terms in ‘culinary nutrition’ and ‘culinary medicine’ are used by practitioners, academics, and researchers and commence the creation of consensus definitions.

## 2. Method

Constructivism was the research paradigm for this study. This view acknowledges that new understanding is socially constructed by researchers through the active interpretation of new data within the context of their pre-existing knowledge and understanding of the topic [39]. The researchers bring with them their knowledge, understanding, and experiences. It is therefore important to recognize the values and stance of the investigators to build internal coherence in research [40].

The investigators involved in this study included academics and researchers in food, nutrition and dietetic practice, and food, nutrition, dietetics, and teaching kitchen practitioners in Australia, Canada, and the United States of America. All investigators had experience in food, nutrition, and the health education of community members, and five of the six had experience in health professional education including nutrition scientists, dietitians, doctors, and other allied health professions.

Leading practitioners, academics, and researchers working in the field of food, nutrition, and health were invited by the investigators to participate in this research. A purposive sampling strategy was initially employed to ensure a wide participant base and to increase the credibility of the results. Purposive sampling is often used in qualitative research; expert purposive sampling is useful when investigating new areas of research and to determine the need for further research [41]. The intention was to collect definitions from individuals known for their engagement in food, nutrition, and health education, in a variety of work settings and locations. This approach was adopted from Johnson et al. [42], who used a purposive and politically important case sampling frame for their work on building consensus for the term ‘mixed methods research’.

Thirty potential participants from Australia, the United States, Canada, United Kingdom, Europe, and Asia were approached via email to submit definitions to key terms including culinary nutrition, culinary nutrition science, culinary medicine, culinary nutrition professional, culinary nutrition intervention, culinary nutrition activity, and culinary nutrition competency via a Qualtrics survey link. Consent was implicit upon entering the survey and completing any definitions. Participants were informed that their name and position relevant to their field of work may be included in the publication of the research findings.

Participants were asked if they were willing to contribute to further discussion once the analysis was completed and to forward the research information and survey link to other practitioners/academics/researchers to participate. This use of the snowball technique to increase the sample size is also used frequently in qualitative research and takes advantage of participant networks and referral [43]. Initial emails were sent in October/November 2021 with two email reminders sent between November 2021 and March 2022. Data were downloaded from Qualtrics in August 2022 on an Excel spreadsheet and definitions for each term moved to individual sheets for analysis.

The investigators chose qualitative content analysis as the research tool to generate draft consensus definitions from the participants’ texts for each term. Content analysis is an approach to derive meaning from focus-group interviews, open-ended questions, observations or fieldwork notes, or any other data collection method that produces text [44]. Analysis was conducted between August and October 2022 by two researchers (SC and OT), with a third available to discuss disputes (ES), based on a simplified version of the process as described by Kleinheksel et al. [45]. Definitions submitted by respondents varied in length with the median between 14 and 21.5 words. Given the brevity of text answers, the process was adapted as outlined in Table 1.

Both researchers worked independently to become familiar with the texts through repeated reading and created units of meaning: 2–5 words that captured individual ideas or concepts within each submitted definition. Codes (typically 1–5 words) were created from the units of meaning, discussed, and finalized into a coding book. Both researchers then allocated one or more codes to each individual definition. The two researchers independently created categories or themes where related codes were organized into short sentences (typically 5–10 words). Consensus was established through further discussion. Draft definitions of terms were created from the categories and shared with the broader research team to determine the final definitions. The research team proposed a conceptual map of the interrelationship of terms.

The Checklist for Authors and Reviewers of Qualitative Research (Appendix A) was used to guide the reporting of this study [46]. This research was approved by the Australian Catholic University Human Research Ethics Committee (2020-3E).

## 3. Results

Thirty-seven participants commenced the survey by entering their details, and twenty-two included one or more definitions for terms. Reasons for non-participation were unclear due to the research method employed. Roles as described by the participants are summarized in Table 2, and Table 3 describes the areas of work or research that the participants identified (note many participants indicated more than one area)**.**

An example of the analysis process steps of the creation of units and codes for the term culinary nutrition is provided in Table 4. Table 5 includes the proposed definitions for each term.

**Figure 1:**
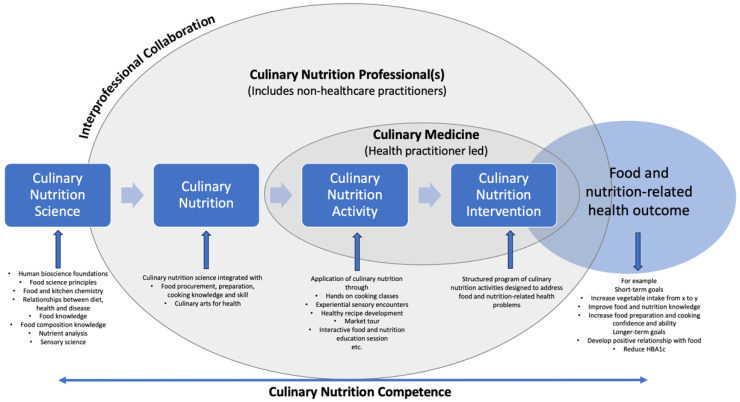
Proposed introductory model of the interrelationship between culinary nutrition terms.

Following a reflection and discussion of the definitions, and to start building an understanding of the interrelationship between the terms, the research team proposed that the terms be divided into two broad categories: practice concepts and the practitioners involved. The concept of culinary nutrition science underpins all other concepts and directly informs culinary nutrition, which is made up of culinary nutrition activities as part of the larger culinary nutrition intervention. To practice culinary nutrition, professionals need to be experts in the translation of the scientific principles and practices of food and nutrition, that is, they have either an individual or collective ability to use the culinary arts and nutrition science together for improved food and nutrition-related health outcomes. Nutrition and health goals are articulated through culinary nutrition interventions, which are in turn supported through activities (e.g., a culinary nutrition intervention goal to ‘increase participant capability to procure, prepare, cook, and consume recommended daily intake of vegetables’ may be supported through culinary nutrition activities such as structured market visits, food budget planning, and hands-on food preparation and cooking classes). The practitioners, culinary nutrition professional(s), engage in the delivery of interprofessional culinary nutrition to improve health outcomes. The breadth of knowledge and skills necessary to implement culinary nutrition activities and interventions requires competence across a range of food and nutrition domains including culinary arts, food and nutrition science, and education, and is typically delivered through teams from diverse professions. As the field continues to mature, it is possible that individual practitioners will develop competencies across multiple domains. However, an important distinction that was apparent through the analysis of the data was when culinary nutrition activities and interventions were delivered by health professionals rather than other professionals (e.g., chefs and/or practitioners) with cooking expertise only. The term culinary medicine therefore describes culinary nutrition delivered by health-practitioners (e.g., medical practitioners, nurses, dietitians). Figure 1 provides a visual map of the proposed interrelationship between terms. Inputs are left to right, culminating in outcomes, however, it is important to note that in practice, program planners start at the right and work to the left (i.e., goals are determined before interventions and activities are planned, and then the appropriate interprofessional team members with the requisite expertise are identified). The model includes examples of elements that make up practice concepts and food and nutrition-related health outcomes, however, it should be noted that these are not exhaustive and can be modified for different practice settings.

## 4. Discussion

This paper describes a wide and diverse interest in culinary nutrition and culinary medicine across different health and paraprofessionals. Despite proliferation in the published literature on this emerging field, there is a lack of consensus in common terms; to the authors’ knowledge, this study was the first to investigate and propose definitions for this field.

Interprofessional health care practice has been established as essential to meet complex health needs with efforts to strengthen IPE for medical and health students [47,48]. This study found a clear consensus that culinary nutrition and culinary medicine should be underpinned by IPP. The proposed definition for culinary nutrition professional(s) is an expert in one or more of the culinary arts, nutrition science, health, and education fields, who takes an interprofessional approach to culinary nutrition. Medical professionals and dietitians are a natural fit, and there are already innovative medical student nutrition education models based on IPE within culinary medicine using teaching kitchens and dietitians as educators [15].

Scope of practice, competencies, professional identities, and role clarity is important for optimization of the health system and quality services [49]. Recognizing that not only health professionals, but also paraprofessionals including chefs [14] are involved in culinary nutrition and culinary medicine, this research proposes a model of interrelationship of concepts and practitioners. Culinary nutrition professional(s) are practitioners who may be health care professionals but are also chefs, health coaches, nutritionists, home economists, and others who meet certain competencies and work interprofessionally with health care professionals as a team to deliver culinary nutrition activities or culinary nutrition interventions. Culinary medicine, however, is a health practitioner-led culinary nutrition activity or culinary nutrition intervention that must be co-designed, co-delivered, and supervised by qualified health care practitioner(s) (e.g., medical practitioner, dietitian, speech pathologist), or part of a shared care model.

In global practice, there are countries with clear regulations on health professional and disciplinary credentialling such as those on the functions of registered dietitian nutritionists, however, in other regions, these are still in development [50]. In the past fifty years, there has been a strong link formed, however, between dietetic leaders from diverse countries through international organizations such as the International Confederation of Dietetics Associations (ICDA) and the European Federation of the Associations of Dietitians (EFAD) [51]. The researchers recognize the widespread practice of culinary and nutrition activities aimed at healthy eating across various populations, irrespective of the formal education or profession of the facilitators. This work further supports the needs for consensus at the level of professional groups in the incorporation of these terms and sets a precedence for individual countries to consider the scope of practice, health care ethics, duty of care, and safety carefully in this emerging field.

The important work in this paper is a crucial and significant first step toward defining terms in culinary nutrition and culinary medicine. The research warrants further development; contributing participants, plus other primary and secondary sources, will be invited to further examine these draft definitions to expand on them and build broader consensus.

As well as individual participants, this process of the evolution of these indicative terms will benefit from consultation within professional and interprofessional groups and organizations at a global level using appropriate research methods. The Delphi research technique is one example of a research method that could be applied. It is often used in health sciences to form consensus through structured group communication processes in which complex issues where knowledge is uncertain and incomplete are evaluated by experts using an iterative process [52]. The authors acknowledge the extensive work ahead to not only reach consensus within disciplines and professions, but also within interprofessional models, frameworks, and agreements to inform a more complete and complex conceptualization of terms for effective education and practice. There are existing examples of the Delphi technique being used to inform competencies in interprofessional practice [53]. As a leadership organization in the field of food is medicine, the Teaching Kitchen Collaborative is well placed to further support research and advocate for global consistencies including through the annual Teaching Kitchen Research Conference [54]. The Teaching Kitchen Collaborative is also the host of an innovative global tool that identifies relevant parties, The Food is Medicine Map, which tracks teaching kitchen initiatives globally and was originally developed by Food at Google, the Lexicon of Sustainability, and thought leaders from the Food Is Medicine Community [54].

Global consistency in the use of terms is important in education and practice, but also for government and other funders, and in developing a robust research and evidence base. Culinary nutrition and culinary medicine research and funding is expanding, particularly within the larger context of food is medicine (also called food as medicine) efforts. The United States of America National Institute of Health recently issued a request for information on Food Is Medicine Research Opportunities, defining “food is medicine services and activities [that] include …culinary medicine and teaching kitchen programs” [55]. Further development of a consistent definition of terms from this study will serve national and global health strategies. Expansion of the definitions of terms should also encompass and complement related definitions, for example, ‘food literacy’ from the scientific literature [56], plus established organizational terms, for example, ‘teaching kitchen’, defined by The Teaching Kitchen Collaborative [54].

The researchers acknowledge that the draft terms are not exhaustive nor potentially representative of all those experienced in this emerging field, and that further development and consultation is required. The declaration that participant names may be published alongside definitions might have deterred some participants from engaging with the study. In addition, the researchers’ credentials and experience may have resulted in inherent bias in understanding historical frameworks, interpreting results, and proposing a draft definition of terms.

Future directions for this work include the development of culinary nutrition competencies and a framework or model for interprofessional best practice. The development of further higher education and professional development opportunities for medical and health students as well as all disciplines involved in culinary nutrition and culinary medicine, will meet the training demands. Ultimately, these developments will assist with global innovation, collaboration, and research and establish a distinct field of interprofessional culinary nutrition and culinary medicine with demonstrated evidenced-based health impacts.

## 5. Conclusions

This paper proposes consensus definitions for common terms used in this fast-evolving area of education and practice. An introductory model of the interrelationship between terms provides a visual map for the development of practice that includes concepts and practitioners both of which come together through interprofessional collaborations to deliver programs to achieve food and nutrition-related health outcomes. Future research should continue to broaden and expand consensus of terms and practices.

## Figures and Tables

**Table 1:** Adapted data analysis process.

Qualitative Content Analysis Process as Outline in Kleinheksel et al. [45]	Adapted Processed Used in This Research
Immersion in the data—becoming intimately familiar with the content	Immersion in the data
Unit of meaning—short description of single concept	Creation of units of meaning
Condensation—shortening unit of meaning while retaining meaning	Creation of codes
Code—1–3 words used to label unit of meaning or condensed unit	
Category—grouping codes with similar meaning (large data sets may require sub-categories)	Creation of categories or themes
Theme—grouping categories to describe behaviors, experience, or emotions across several categories	

**Table 2:** Participant roles.

Role	Number
Academic	7
Dietitian	8
Medical practitioner	2
Nurse	1
Educator (chef, nutrition, food)	3
Food and wine writer	1

**Table 3:** Participant areas of work/research.

Areas of Work/Research	Number
Food/culinary history	1
Culinary nutrition	4
Culinary medicine	5
Writing	2
Tertiary education	2
Pediatric medicine	1
Clinical dietetic practice	2
Food/nutrition education	4
Research	4
Chef education	1
Public health	3
Community health	3
Primary health care	4
Teaching kitchen	4

**Table 4:** Example of the analysis process for the term *culinary nutrition*.

Definition Submitted	Units	Codes
Culinary nutrition represents an understanding of nutrition applied to cooking	Nutrition applied to cooking	Integrating cooking and nutrition Application
Education focusing on healthy cooking for patients	Education Healthy cooking Patients	Healthy food/meals Application
Combining the science of nutrition with demonstration or the hands on practice of food and beverage preparation and consumption	Incorporating nutrition science with food and beverage preparation	Integrating cooking and nutrition Application
The merging of culinary arts and nutrition; knowledge, understanding, and skills to create food and drink that meets the needs of consumers—appeals to all the senses and balances nutrient requirements. Great looking and tasting food that consumers want to eat but also considers the prevailing dietary guidelines as well as specific needs for consumers	Merging culinary arts and nutrition Consumer needs Sensory appeal Meeting nutrient requirements	Integrating cooking and nutrition Nutrition and health goals Sensory appeal Extended culinary arts

**Table 5:** Proposed definitions for terms.

Proposed Definitions	
Culinary nutrition	The integration of culinary arts and nutrition that applies practical knowledge and skills to improve food and nutrition-related health.
Culinary nutrition science	The integration and application of foundation sciences (nutrition, food, cooking science) through culinary nutrition.
Culinary medicine	A health practitioner-led culinary nutrition intervention or activity.
Culinary nutrition professional	An expert in one or more of the culinary arts, nutrition science, health, and education fields, who takes an interprofessional approach to culinary nutrition.
Culinary nutrition intervention	A set of activities to support nutrition and health goals and behavior change in individuals and groups.
Culinary nutrition activity	An experiential educational activity designed to build culinary nutrition competence in individuals and groups.
Culinary nutrition competency	An agreed set of competencies (demonstrated knowledge, understanding and skills in culinary nutrition) for culinary nutrition professionals or participants.

## Data Availability

Data are contained within the article.
